# Characterization of a pathogenic variant in *GBA* for Parkinson’s disease with mild cognitive impairment patients

**DOI:** 10.1186/s13041-020-00637-x

**Published:** 2020-07-08

**Authors:** Zhiqiang Jiang, Yilin Huang, Piao Zhang, Chongyin Han, Yueer Lu, Zongchao Mo, Zhanyu Zhang, Xin Li, Sisi Zhao, Fuqiang Cai, Lizhen Huang, Chunbo Chen, Zhihong Shi, Yuhu Zhang, Fei Ling

**Affiliations:** 1 School of Biology and Biological Engineering, South China University of Technology, Guangzhou, China; 2 Department of Neurology, Guangdong Neuroscience Institute, Guangdong Provincial People’s Hospital, Guangdong Academy of Medical Sciences, No. 106. Zhongshan Er Road, Guangzhou, 510080 Guangdong Province PR China; 3 Department of emergency and critical medicine, Guangdong Provincial People’s Hospital, No. 106. Zhongshan ErRoad, Guangzhou, 510080 Guangdong Province PR China; 4 Tianjin Key Laboratory of Cerebrovascular and Neurodegenerative Diseases, Tianjin Dementia Institute, Department of Neurology, Tianjin Huanhu Hospital, Tianjin, China

**Keywords:** *GBA*, rs12411216, α-Synuclein, Parkinson’s disease-mild cognitive impairment

## Abstract

Parkinson’s disease (PD) is the second most common neurodegenerative disease, and mild cognitive impairment (MCI) is a well-established risk factor for the development of dementia in PD. A growing body of evidence suggests that low expression of glucocerebrosidase (*GBA*) promotes the transmission of α-synuclein (α-Syn) interpolymers and the progression of PD. However, how *GBA* mutations affect the pathogenesis of PD via abnormal aggregation of α-Syn is unclear, and no clinically valid PD-MCI genetic markers have been identified. Here, we first located a *GBA* eQTL, rs12411216, by analysing DHS, eQTL SNP, and transcription factor binding site data using the UCSC database. Subsequently, we found that rs12411216 was significantly associated with PD-MCI (*P* < 0.05) in 306 PD patients by genotyping. In exploring the relationship between rs12411216 and *GBA* expression, the SNP was found to be associated with *GBA* expression in 50 PD patients through qPCR verification. In a further CRISPR/Cas9-mediated genome editing module, the SNP was identified to cause a decrease in *GBA* expression, weaken enzymatic activity and enhance the abnormal aggregation of α-Syn in SH-SY5Y cells. Additionally, using an electrophoretic mobility shift assay, we confirmed that the binding efficiency of transcription factor E2F4 was affected by the rs12411216 SNP. In conclusion, our results showed that rs12411216 regulated GBA expression, supporting its potential role as a PD-MCI genetic biomarker and highlighting novel mechanisms underlying Parkinson’s disease.

## Introduction

Parkinson’s disease (PD) is the second most common degenerative disease after Alzheimer’s disease. It involves the degeneration of dopaminergic neurons in the substantia nigra pars compacta and diffuse Lewy body deposition. The clinical manifestations of PD include resting tremor, bradykinesia, myotonia, abnormal posture and gait disorder. Additionally, this disease is accompanied by non-motor symptoms such as depression, constipation and sleep disorders, which impose a heavy burden on patients, families and society [1]. The motor symptoms of PD included motor retardation, tremor, and myotonia. Both posture and gait abnormalities are well documented, but non-motor symptoms are often overlooked.

Cognitive impairments, including Parkinson’s disease-mild cognitive impairment (MCI) and Parkinson’s disease dementia (PDD), were the most common and disabling non-motor symptoms [2]. MCI is an early warning signal of late dementia in PD. Research has shown that nearly 30% of newly diagnosed PD patients have MCI, more than 40% of PD patients with normal cognitive function will develop MCI within 6 years, and nearly 80% of patients may develop PDD at the late stage of PD [3]. Moreover, the early diagnosis and prevention of PD-MCI can prevent the development of PDD. However, effective clinical genetic markers or targets for early diagnosis are currently not available.

The *GBA*-encoded protein glucocerebrosidase can decompose glucocerebroside into glucose and ceramide. Recent studies showed that low *GBA* expression promoted the prion-like spread of α-Syn interpolymer complexes, facilitated the pathogenesis of PD and increased cognitive damage [3, 4]. *GBA* gene expression was high in the brain tissue of healthy individuals but was decreased in that of PD patients [3, 5]. Genetic and clinical studies confirmed that *GBA* mutations were more prevalent in PD patients than in other populations [6, 7]. Similar to the common N370S and L444P mutations, *GBA* heterozygous mutations that were associated with the onset of PD were defined as deleterious [8, 9]. In most PD populations, the *GBA* mutation frequency was higher than that of other PD-related genes. A study consisting of 5691 PD patients and 4988 controls showed that the odds ratio of *GBA* mutations (L444P & N370S) in PD patients was 5.43 (95% confidence interval: 3.89–7.57), indicating that *GBA* mutations were important factors in PD development [6].

Regardless of ethnicity, age, or sex, PD patients had a higher frequency of *GBA* mutations than the control individuals. In a study containing 99 patients with idiopathic PD from Israel and 1543 Jewish controls from Germany, a significantly higher proportion of Parkinson’s disease patients (31.3%) were found to carry one or more *GBA* mutations than control groups (6.2%, *P* < 0.001) by screening six common *GBA* mutations [10]. Similar findings could be observed from studies of different ethnic groups in other regions [11]. Furthermore, a large US cohort study revealed that the *GBA* mutation frequency in familial PD was 4.1%, while in the control population, only 1.1% of individuals harboured such mutations [6]. Taken together, for both idiopathic and familial Parkinson’s disease, the frequency of *GBA* mutations was higher in case groups than in control populations [12]. Accordingly, a recent study showed that knocking out the *GBA* gene in human neuroblastoma cells (SH-SY5Y cells) led to neuronal glycosphingolipid accumulation [13].

Current studies have shown that abnormal aggregation of α-Syn is a significant pathological feature of PD, and the expression of *SNCA, a gene* encoding α-Syn, is directly related to the pathogenesis of PD [12, 14]. Additionally, the decrease in the proportion of α-Syn tetramers and the increase in the proportion of α-Syn monomers (which can form abnormal aggregates) in cells could easily lead to the onset of PD [13, 15]. Moreover, the loss of *GBA* function increased α-Syn levels, promoted its prion-like spread and aggregation and aggravated cognitive impairment in PD [16, 17]. However, the relationship between *GBA* mutations and abnormal α-Syn aggregation and their roles in the pathogenesis of PD remains unclear.

In this study, by retrieving data from the UCSC database, we hypothesized that the rs12411216 SNP might regulate the functions of the *GBA* gene. The rs12411216 SNP was found to overlap with the core motif of transcription factor E2F4 and served as the candidate for downstream analysis. For our downstream validation, a total of 306 PD outpatients were recruited from two hospitals for cognitive impairment assessment and genotyping. GBA expression, enzymatic activity, and the aggregation of α-Syn were measured in CRISPR/Cas9-edited SH-SY5Y cells. Mechanistically, electrophoretic mobility shift assays (EMSAs) were applied to preliminarily explore declines in transcription factor binding.

## Materials and methods

### Bioinformatics analysis

#### Definition of GBA regulatory regions

Using the NCBI database, we obtained the coordinates of the *GBA* gene in the human genome sequence (hg19 version). The regulatory regions of the *GBA* gene were delineated as sequences ±50 kb away from the transcriptional start site.

#### Identification of DHSs potentially regulating GBA

Total DHS data (track: wgEncodeRegDnaseClusteredV3) were obtained from the UCSC website. All these data were converted to hg19 version using the UCSC liftover tool for consistency. Single base-pair DHS data were removed as they were not recognized in subsequent processing procedures. The intersection of the processed DHS data was retained, and regulatory regions of the *GBA* gene were decided using BEDOPS with the “bedops -element-of 1” parameter. Of these identified DHSs, those that reacted to the GBA promoter were selected according to Hi-C data, which were obtained from a previous study [18]. A total of 143 DHSs located in the regulatory regions of *GBA* were identified and termed *GBA*-related DHSs that potentially regulated GBA expression.

#### Biological analysis of rs12411216 regulatory function

eQTL data (filename: gtexEqtlCluster) were downloaded from the UCSC website (version hg19. 8), and eQTL SNPs lying within *GBA*-related DHSs were selected as primary candidates. To examine their regulatory function in *GBA*, after searching against the transcription factor binding site database (filename: wgEncodeRegTfbsClustered), the rs12411216 SNP was found to overlap with the core motif of transcription factor E2F4 and served as the candidate for downstream analysis.

### Participants

A total of 306 PD patients from Guangdong Provincial People's Hospital (259 patients) and Tianjin Huanhu Hospital (47 patients) underwent an rs12411216 SNP typing test and a “PD-NC&PD-MCI typing test”. Global cognitive and functional status was assessed using the Mini-Mental State Examination (MMSE) and the Montreal Cognitive Assessment (MOCA). All the patients were assessed with the Animal Fluency Test and Picture Arrangement Test (Wechsler Adult Intelligence Scale, WAIS-R) for executive functions, the Digit Span and Digit Symbol tests for attention and working memory, the Logical Memory Test and Immediate Memory Test (Wechsler Memory Scale WMS) for memory, the Vocabulary and Similarities Test (WAIS-R) for language, and the Block Design and Object Assembly tests for visuospatial function.

Patients with PD were divided into those with normal cognition (PD-NC) and those with mild cognitive impairment (PD-MCI) according to the Level II criteria of the 2012 Movement Disorder Society (MDS) Task Force Guidelines. A total of 50 PD patients from Guangdong Provincial People's Hospital underwent genotyping for the rs12411216 SNP and the *GBA* expression test.

### CRISPR/Cas9-mediated knockout of the DHS and the replacement of rs12411216

Based on the DNase hypersensitive site (DHS) sequence of PD (chr12: 131621602–131,623,539), we designed single guide RNAs (sgRNAs) to knock out this regulatory region in the SH-SY5Y cell line. pCMV-Cas9, a vector that has a selectable neomycin marker, was obtained from Addgene (41815). The following sgRNAs were designed via the Zhang Laboratory website (https://zlab.bio/guide-design-resources) for the DHS-knockout experiment: sgRNA1, 5′-GGAGTGCAATGGCGCGATCTCGG-3′ and sgRNA2, 5′- CTCCGTCTCAAAACAAAAAAGGG-3′. The following sgRNA was designed for the *GBA*-SNP replacement experiment: SNP-sgRNA: GTGCGGCGAGACCCTGGGGCAGG, along with donor DNA (Supplementary Material). These sgRNAs were then cloned into U6-sgRNA plasmid vectors. A total of 1 μg of DNA (0.25 μg sgRNA1 vector or SNP-sgRNA vector + 0.25 μg sgRNA2 vector or donor DNA vector + 0.5 μg pCMV-Cas9-vector) was diluted in 50 μl Dulbecco’s modified Eagle’s medium (DMEM). Next, 3 μl GenJet In Vitro DNA Transfection Reagent (SL100489; SignaGen Laboratories) was diluted in 50 μl Dulbecco’s modified Eagle’s medium (DMEM), and two solutions were mixed and incubated for 20 min. After that, sgRNA1, sgRNA2 and pCMV-Cas9 vectors (0.25 μg sgRNA1 vector + 0.25 μg sgRNA2 vector + 0.5 μg pCMV-Cas9-vector) were cotransfected into the SH-SY5Y cell line using GenJet In Vitro DNA Transfection Reagent. To select cells expressing the neomycin resistance protein, the antibiotic G418 (800 μg/ml) was added to the medium and DMEM 48 h after the transfection, with G418 thereafter replaced every 48 h. After the addition of G418 for 8 days, single colonies were selected using the limiting dilution and verified by both PCR and sequencing methods [19].

### GCase enzyme activity assay

GCase enzyme activity was assessed using the following methods. First, enriched lysosomes in 50 μl of activity assay buffer were added to a 96-well plate. This composition of this buffer was 0.25% Triton X-100 (TX; Sigma-Aldrich), 0.25% taurocholic acid (Sigma-Aldrich) and 1 mM ethylenediaminetetraacetic acid (EDTA) in citrate-phosphate buffer (pH 5.4). Second, 50 μl of 1% bovine serum albumin with 1 mM of the GBA substrate 4-methylumbelliferyl β-glucoside (M3633; Sigma-Aldrich) and/or 10 mM of the GBA inhibitor conduritol B epoxide (Sigma-Aldrich) were added, followed by incubation at 37 °C for 40 min. Last, 50 μl of 1 M glycine (pH 12.5) was added to terminate the reaction, and the fluorescence was measured using a plate reader (excitation = 355 nm, emission = 460 nm, 0.1 s; PerkinElmer) [13].

### Electrophoretic mobility shift assay (EMSA)

First, nuclear extract was isolated from SH-SY5Y cells using Biyuntian kit P0028. Second, 3 μg of nucleoprotein was mixed with a biotin-labelled probe (Supplementary Material) containing rs12411216 (50 fmol) and incubated for 20 min at 25 °C. Third, the mixture (some of which was attached to the biotin-labelled probe) was separated using 6% polyacrylamide gel in Tris-glycine-EDTA running buffer at 4 °C for 2 h. Last, molecules attached on the gel were then transferred and assessed using an enhanced chemiluminescence detection system (Sage Creation, Beijing, China) [20].

### Immunostaining analysis

We first placed SH-SY5Y cells on coverslips with poly-D-lysine coating. Next, 4% paraformaldehyde was used to fix cells, followed by blocking with 5% normal donkey serum (Jackson ImmunoResearch, Bar Harbor, ME, USA) and 0.1% TX (Sigma-Aldrich) for 1 h at room temperature. To assess the expression levels of p-α-Syn, a series of incubations with the primary antibody anti-p-α-Syn (1:1000, Abcam) were conducted at 4 °C overnight. The samples were washed off with 0.1% TX in phosphate-buffered saline, followed by 1 h of incubation with a mixture of fluorescein isothiocyanate (FITC)-conjugated secondary antibody (Jackson ImmunoResearch Laboratories, Inc.) and cyanine 3 (CY3)-conjugated secondary antibody (Jackson ImmunoResearch Laboratories, Inc.) at room temperature. Fluorescent images were acquired using a confocal microscope (Zeiss Confocal LSM 710) after coverslips were mounted. All images were processed using Zeiss Zen software. The range of signal intensity of the threshold in the selected areas was measured using ImageJ software.

### Quantitative PCR (qPCR)

*GBA* and *SNCA* primer pairs are listed in the Supplementary Material. The β-actin gene was used as a reference gene for the qPCR analysis. Reagents for qPCR were obtained from Takara Biotechnology (DRR096A; Dalian, China). Expression data of GBA and SNCA genes in both wild-type SH-SY5Y (eight replicates) and knockout SH-SY5Y cells (six replicates) were collected for downstream analysis. Relative expression was calculated using the following formula: relative expression = 2^−ΔΔCt^; the relative expression was normalized based on the expression level of knockout SH-SY5Y cells. *P*-values for the differences between the wild-type and knockout cell groups were calculated using the t-test.

### Western blotting

SH-SY5Y cells were lysed in 300 μl of radioimmunoprecipitation assay buffer (Biotech Well; Shanghai, China) in a 96-well plate containing 1 mM phenylmethylsulfonyl fluoride (PMSF) per well. Total protein concentrations were determined by bicinchoninic acid assays (Biotech Well). Samples were normalized with lysis buffer to ensure that each sample had the same protein concentration. Next, samples were mixed with an equal volume of 2× Laemmli sample buffer and solubilized by boiling for 10 min at 99 °C. Proteins were separated by sodium dodecyl sulfate polyacrylamide gel electrophoresis (SDS-PAGE), and the proteins were then detected using mouse monoclonal antibodies against GBA, α-Syn and phospho-Ser129 α-Syn (p-α-Syn, a marker of pathologic α-Syn) at the dilution recommended by the manufacturer (EarthOx, San Francisco, CA, USA). β-actin was detected using a mouse monoclonal antibody against β-actin (EarthOx, San Francisco, CA, USA).

### Statistical analysis

The chi-squared test was used to verify the association between rs12411216 and PD-MCI in clinical samples. The data are depicted as means ± standard deviations and were analysed using an unpaired Student’s t-test. A *P* value smaller than 0.05 was defined as statistically significant. All graphs and statistical calculations were performed using GraphPad Prism (Version 6.0). Other supplementary materials were available online.

## Results

### The rs12411216 SNP was significantly associated with PD-MCI

**Fig. 1:**
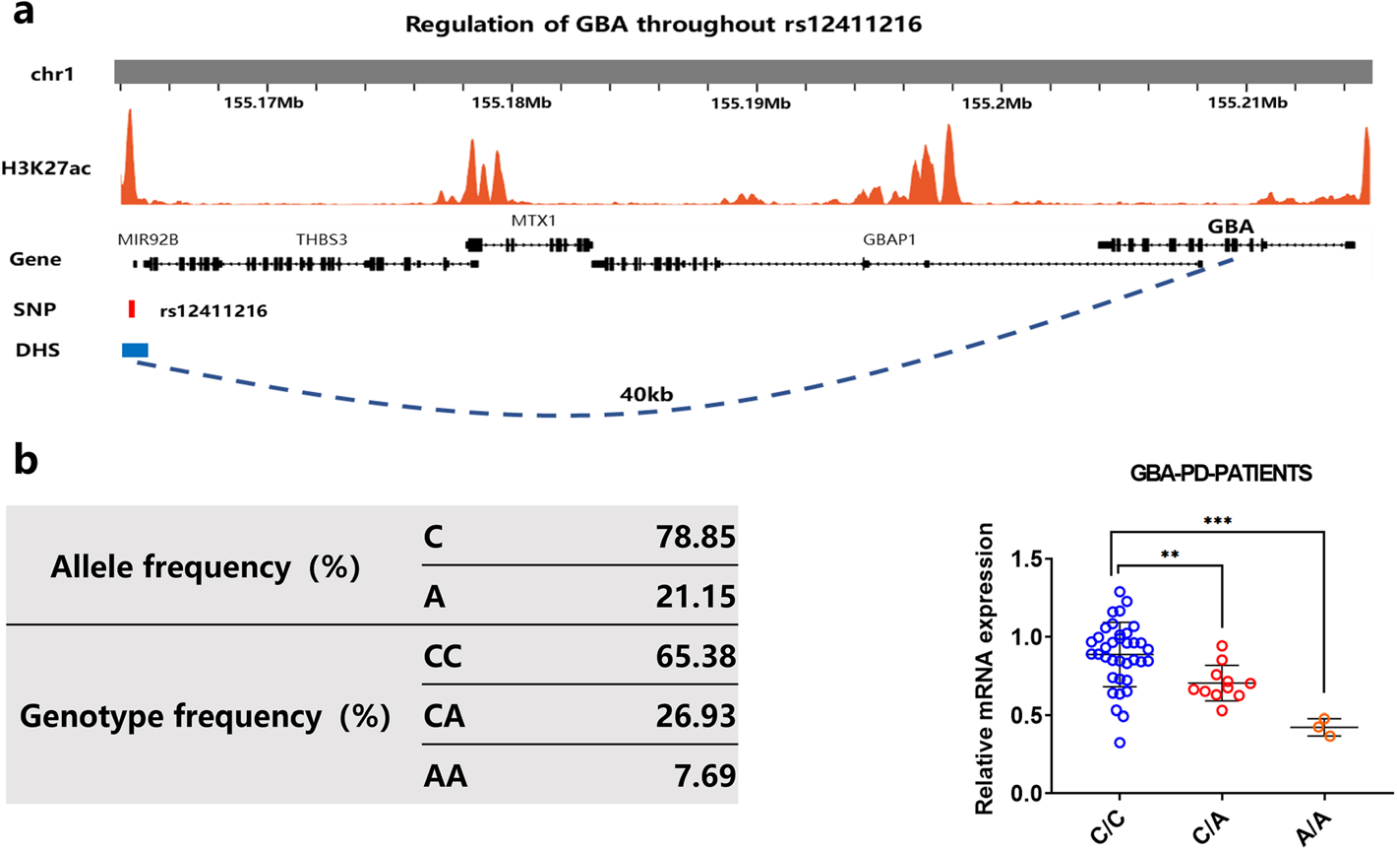
SNP phenotypic variation in clinical samples, with changes in *GBA* expression. **a** Screening process to identify rs12411216 and related information in the UCSC database. Based on Hi-C data, a DHS (with *GBA* as the potential target gene) was found, and an eQTL affecting *GBA* expression was found in the DHS. **b** Genotyping of rs12411216 and the expression of *GBA* mRNA in clinical samples from patients with Parkinson’s disease

In exploring the presence of a regulatory SNP of the GBA gene, the eQTL SNP rs12411216 was identified as a candidate in the distal regulatory element of the *GBA* gene using the UCSC database (Fig. 1a). Allelic and genotypic comparisons between 122 PD-MCI patients and 184 PD-NC patients who had Parkinson’s disease but no cognitive impairment showed that 5.56% (*n* = 17) of PD patients (*n* = 306) had PD-MCI and a homozygous (A/A) rs12411216 genotype, while 16.67% (*n* = 51) had PD-MCI and a heterozygous (C/A) rs12411216 genotype. The allele frequencies among the PD-MCI patients were 34.84% for A and 65.16% for C and were consistent with the 1000 Genomes Project datasets. The rs12411216 genotype was significantly associated with PD-MCI (*chi*-squared test, *P* = 0.0478).

### The rs12411216 SNP was significantly associated with *GBA* expression in PD patients

After genotyping 50 PD patients from Guangdong Provincial People's Hospital, we found that, consistent with the 1000 Genomes Project data, GBA allele frequencies were 72.00% for C/C, 22.00% for C/A, and 6.00% for A/A. Additionally, the *GBA* mRNA level was significantly decreased in patients with homozygous mutations (AA genotype) compared to patients with the wild-type genotype (CC) (Fig. 1b).

### The rs12411216 SNP affected the expression of *GBA* by decreasing the binding efficiency of transcription factor E2F4

**Fig. 2:**
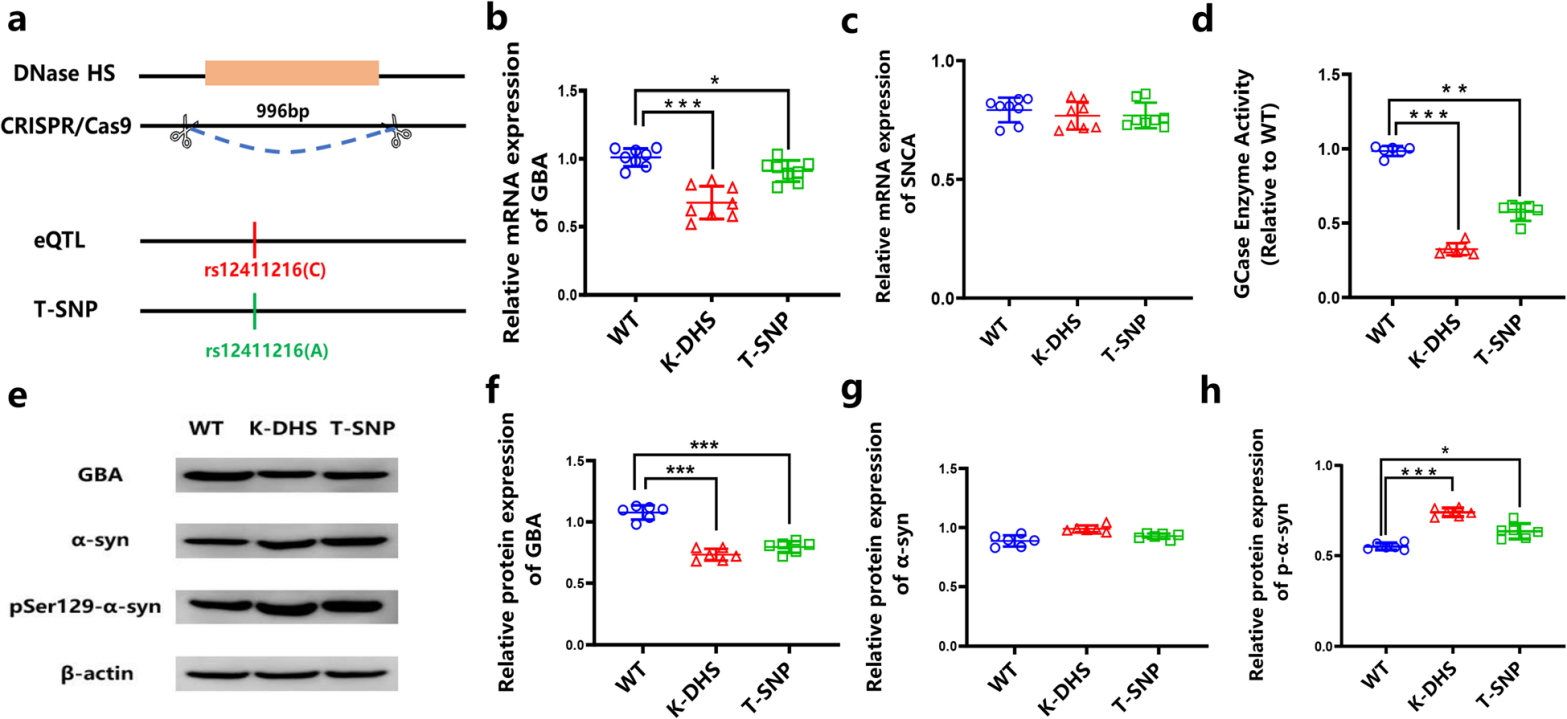
The rs12411216 SNP affects *GBA* expression. **a** Positions in the genome of the knocked out DHS and the replaced SNP. **b**, **c** mRNA levels of *GBA* and *SNCA*. **d** GBA enzymatic activity (normalized to the control) measured using lysosome-enriched fractions in control SH-SY5Y cells, DHS-knockout SH-SY5Y cells and SNP-replaced SH-SY5Y cells (*n* = 6). **e–h** Western blotting results before and after DHS knockout and SNP replacement. *p < 0.05; ** *p* < 0.01; ***p < 0.001. Data are expressed as means ± SEMs. K-DHS: DHS-knockout cells; T-SNP: SNP-replaced cells

To verify whether the rs12411216 SNP affected *GBA* expression in vivo, the rs12411216 SNP was replaced, and the upstream DHS sequence of *GBA* (chr1: 155164145–155,164,895) was knocked out in SH-SY5Y cells using the CRISPR/Cas9-mediated genome editing method (Fig. 2a). Our results showed that the DHS sequence significantly affected the expression of *GBA* (Fig. 2b–c), and rs12411216 was a functional SNP of the *GBA* gene (Fig. 2e–g). To determine whether *GBA* mutation affected the expression of *SNCA*, an important gene implicated in PD, we investigated the mRNA expression of *SNCA* and the protein expression of α-Syn and p-α-Syn in the knockout and replacement cells (Fig. 2h). We found no significant difference in the mRNA or protein expression of the *SNCA* gene, but the level of p-α-Syn was significantly increased both in the knockout and in the replacement cells. Together, these results indicated that rs12411216 was a regulatory SNP of the *GBA* gene and that the DHS fragment might serve as a regulatory element affecting the expression of the *GBA* gene.

**Fig. 3:**
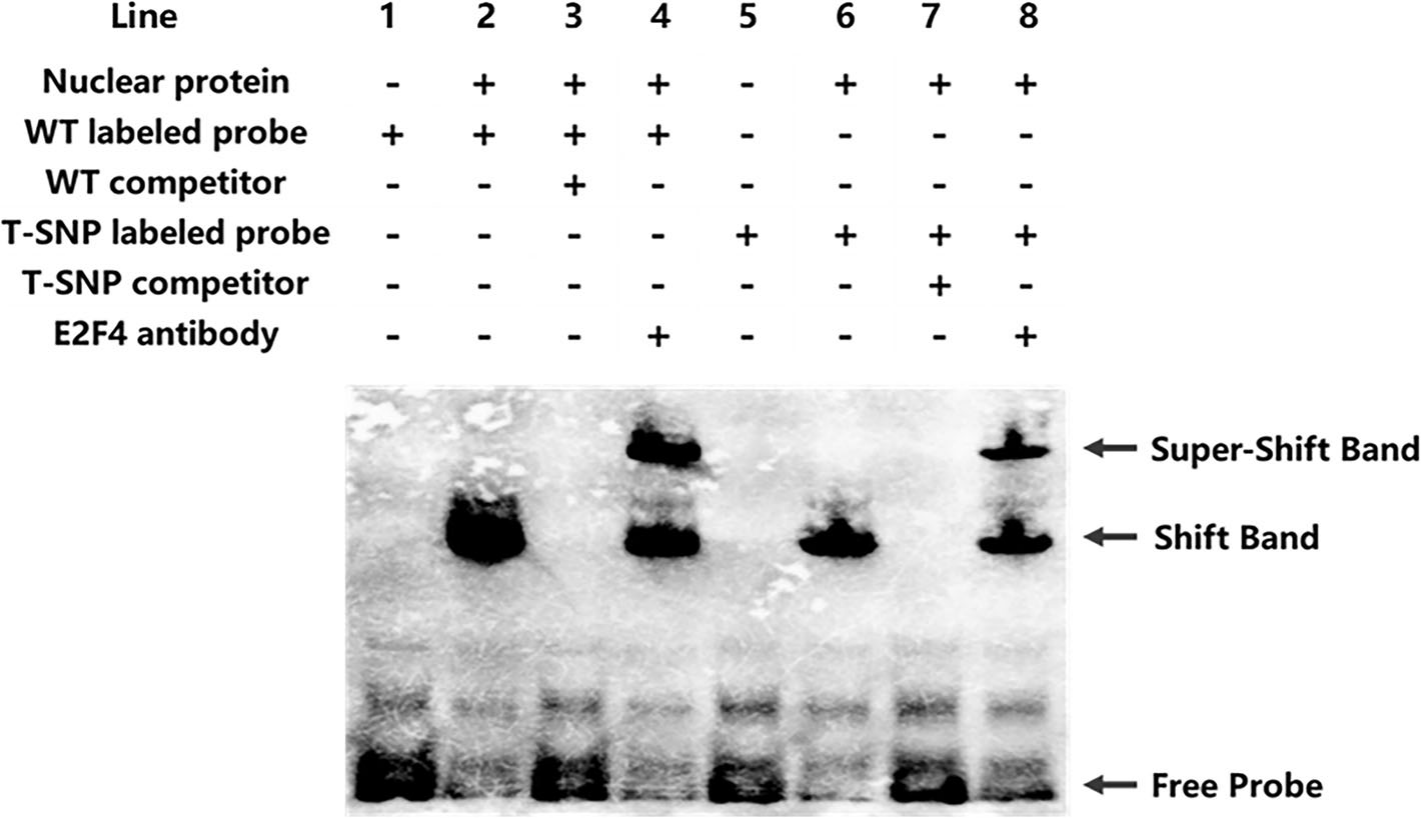
The rs12411216 SNP mutation decreased the binding activity of transcription factor E2F4. WT labelled probe: wild-type SNP biotin-labelled probe; T-SNP labelled probe: mutant SNP biotin-labelled probe; Competitors: a large number of biotin-free markers. The EMSA demonstrated that the WT probe, T-SNP probe, complexes of probes and E2F4 antibody could bind to the nuclear extract. The rs12411216 mutation decreased the binding ability of transcription factor E2F4

To uncover how the regulatory SNP affects the expression of the *GBA* gene, we further analysed the presence of a core binding motif of transcription factor E2F4 within this DHS fragment. Based on the EMSA, we observed that when the wild-type SNP biotin-labelled probe (rs12411216: C) was applied, the probe bound to the nuclear protein, and a shifted band could be observed (Fig. 3, Line 2). After adding E2F4 antibody, a super-shift band could also be observed (Fig. 3, Line 4). However, when using the mutant SNP biotin-labelled probe (rs12411216: A), the binding ability of the probe to the nuclear protein was much weaker (Fig. 3, Line 6) than that of the wild-type biotin-labelled probe, and the binding ability to E2F4 was also reduced (Fig. 3, Line 8). In conclusion, our results showed that the rs12411216 mutation decreased the binding capacity of transcription factor E2F4.

### rs12411216 affects GCase enzyme activity and α-Syn aggregation

**Fig. 4:**
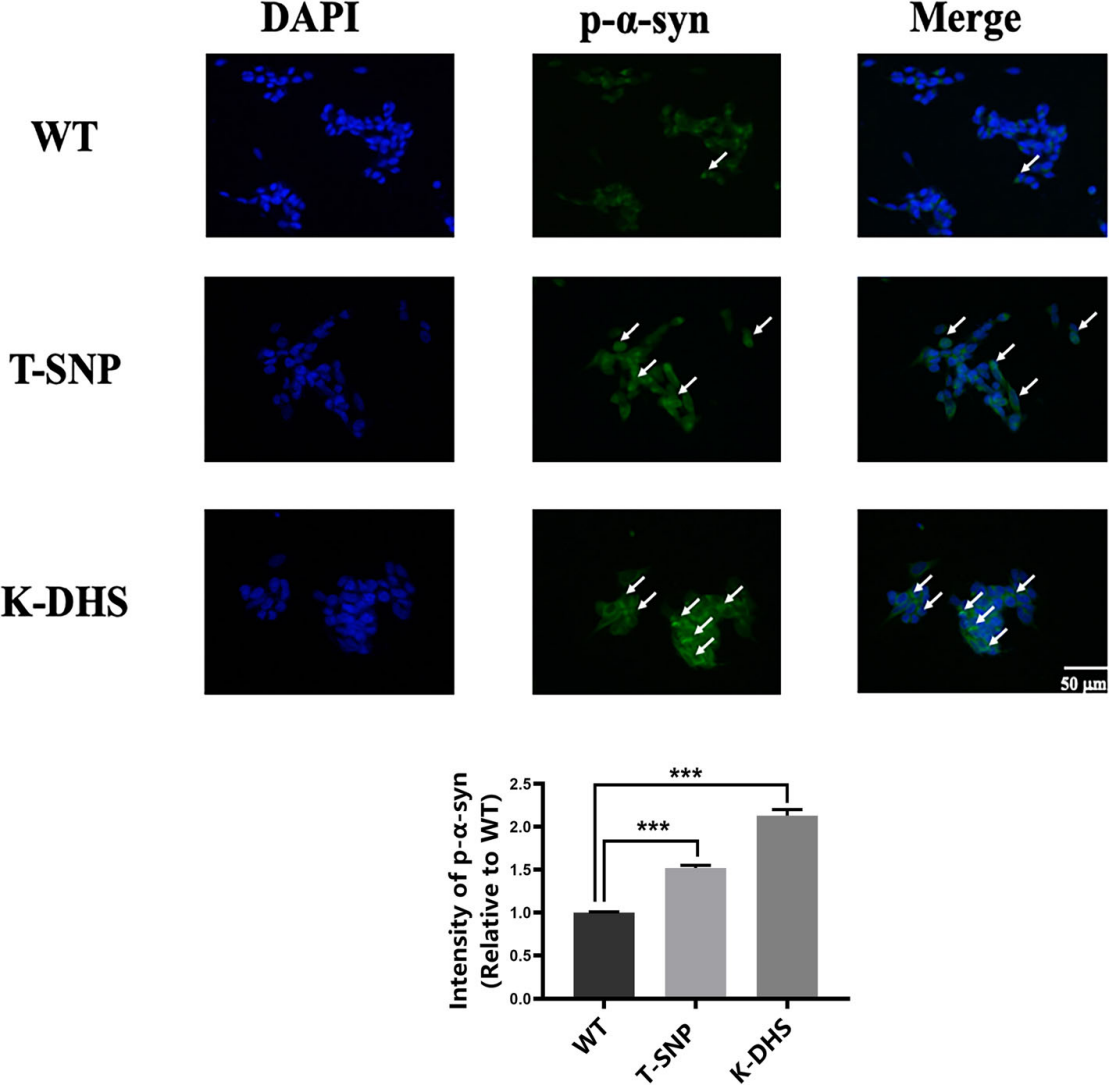
The rs12411216 SNP affects the aggregation of p-α-syn. K-DHS: DHS-knockout cells; T-SNP: SNP-replaced cells. Blue fluorescence represents 4′,6-diamidino-2-phenylindole (DAPI)-stained nuclei, and green fluorescence represents p-α-Syn expression. Immunofluorescence experiments indicated that rs12411216 affected p-α-Syn aggregation. ***p < 0.001. Data are expressed as means ± SEMs

As shown in Fig. 2d, GCase enzyme activity was significantly decreased in both the DHS knockout and rs12411216 replacement cells compared to that in the wild-type cells. By using anti-p-Syn antibody in a cellular immunofluorescence analysis, we observed that intracellular p-Syn levels were increased by 2.13- and 1.52-fold after the knockout of the DHS and the replacement of the SNP, respectively (Fig. 4). The pathological aggregation of α-Syn was more severe in cells with rs12411216 replacement. These results indicated that replacing rs12411216 could cause pathological aggregation of α-Syn and increase the risk of PD.

## Discussion

This study chose the *GBA* gene, because mutations in the *GBA* gene are recognized as a strong risk factor for the development of PD and dementia with Lewy bodies (DLB). The reason for choosing the rs1241126 SNP was that the rs12411216 SNP is located near the core binding domain of transcription factor E2F4 in the regulatory element of the *GBA* gene (the corresponding DHS).

Our screening process and specific criteria for the DHS and SNP have been added to the discussion section, and the details are listed as follows. In this study, DNase-seq data in the UCSC database were used to obtain chromatin opening (DHSs) sites of Parkinson’s disease-related genes. These DHSs may regulate the expression of Parkinson’s disease-related genes (such as *SNCA* and *GBA*). Though the DHS data revealed several potential regulatory regions of *GBA*, they cannot directly suggest the phenotypic changes. Therefore, we filtered the DHS data combining Hi-C data and eQTL SNP data, which provide chromatin reaction data and the connection between SNPs and gene expression.

First, after mining the Hi-C data of Parkinson’s disease from the ENCODE database, we obtained the gene locations of chromatin that directly acted on the promoter of Parkinson’s disease. Based on the binding sites screened in the DHS and Hi-C data, we compared eQTL data and found that the rs12411216 SNP was located near the core binding domain of transcription factor E2F4, which was the regulatory element of the *GBA* gene (the corresponding DHS).

Additionally, according to a large number of brain tissue sample expression datasets, mRNAs harbouring this SNP were found to be highly expressed, suggesting that this SNP might be a brain-tissue-specific regulatory marker. Thus, we hypothesized that this SNP mutation might affect the binding of transcription factors to both enhancers and promoters by changing the binding efficiency, affecting the expression of the *GBA* gene as a result.

Next, we recruited 306 Parkinson’s patients (including PD-NC & PD-MCI based on MMSE and MOCA scores) and determined their cognitive impairment status. After genotyping, we found that the rs12411216 SNP was marginally significantly associated with PD-MCI (*P* = 0.0478). Subsequently, by using both in vivo and in vitro assays, we demonstrated that the rs12411216 SNP could decrease *GBA* expression, enhance abnormal aggregation of α-Syn, and affect the binding efficiency of transcription factor E2F4.

Our results of 306 PD patients showed that the rs12411216 SNP was significantly associated with PD-MCI. However, the *P*-value (*P* = 0.0478) for such an association is near the borderline, which was likely due to the small sample size in this study. In another independent study in which 790 PD patients were genotyped for the association of GBA variants with PD, PD patients with GBA mutations had a higher frequency of cognitive dysfunction than non-carriers [21]. Considering these findings, we concluded that the rs12411216 SNP is a functional variant that can serve as a genetic marker for PD-MCI.

Interestingly, our quantitative expression analysis revealed that PD patients (*n* = 3) with homozygous mutations (AA genotype) had lower GBA expression than patients with the other two genotypes (i.e., CA or CC). Of these three patients, one underwent cognitive impairment assessment (mainly using the MMSE and MoCA) and was identified to have PD-MCI. In addition, more studies demonstrated that *GBA* mutations were significantly associated with PD-MCI [3, 22], indicating that PD patients with the AA genotype for the rs12411216 SNP were more likely to develop PD-MCI.

Moreover, a recent GWAS study found a significant association between the rs12411216 SNP and occipital lobe volume (*P*_European ancestry-only_ = 3.9 × 10^− 8^) [23]. The occipital lobe is part of the cerebral cortex and is related to cognitive ability. Using neuroimaging tests, some studies have reported that PD-MCI patients may develop local brain atrophy (including occipital atrophy) at an early stage [24, 25]. These studies suggested that occipital lobe volume might be a factor in the pathogenesis of PD-MCI.

Studies focusing on *GBA* in PD have pointed out that several processes might be involved in *GBA*-related Parkinson’s disease, including lysosomal dysfunction [26], α-Syn-related mechanisms [27], sphingolipid dysregulation [28], autophagy defects and protein transport defects [29]. However, identifying specific pathways and mechanisms requires further investigation. Therefore, the goal of our future studies is to investigate the potential mechanism of *GBA* in PD. An increasing number of reports confirmed that most disease-related mutations were located in non-coding regulatory regions [30]. Genome editing in human neural progenitors suggested that the distal SNP rs1191151 (downstream 700 kb) regulated FOXG1 expression, supporting its potential role as a schizophrenia risk gene [31]. Our results showed that the DHS of rs12411216 regulates GBA expression despite being located 40 kb upstream of the GBA gene.

**Fig. 5:**
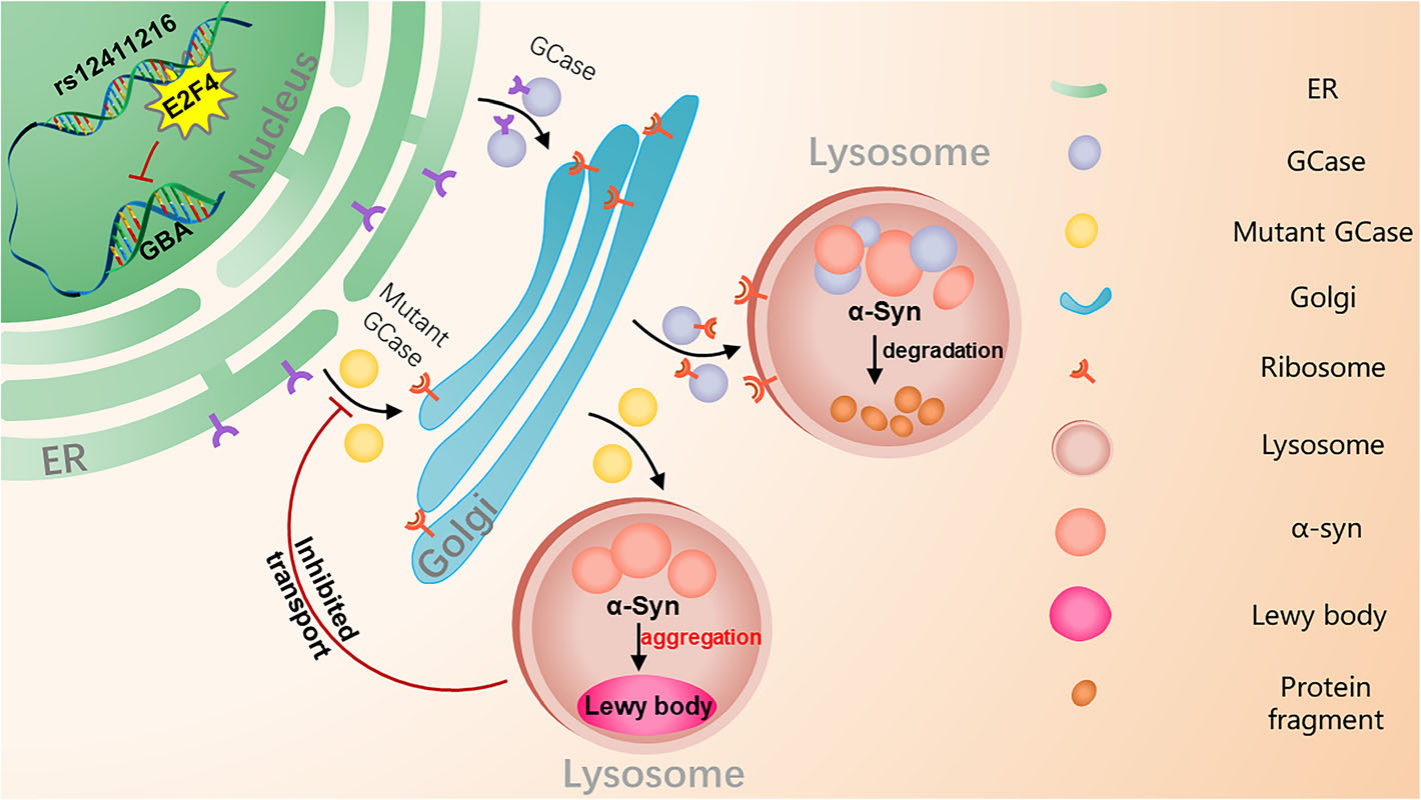
Abstract picture. Under normal conditions, GCase is synthesized in the endoplasmic reticulum (ER)-bound polysomes, from which it is transferred to the ER. After glycosylation, GCase is transported to the Golgi apparatus and then to the lysosome, and α-synuclein is normally degraded. Under abnormal conditions, the mutation of the rs12411216 site leads to a decrease in e2f4 binding efficiency, which will cause a reduction or loss of GCase activity. GCase cannot be transported to the lysosome, causing the accumulation and oligomerization of α-synuclein and eventually the formation of a Lewy body. In turn, elevated α-synuclein inhibits lysosomal maturation and the activity of normal GCase. α-Synuclein prevents the transfer of GCase from the endoplasmic reticulum to the lysosomes

Genome editing and EMSA results showed that the binding activity of E2F4 was weakened, as proposed in our hypothesis. In SH-SY5Y cells, the rs12411216 mutation may not affect the expression of SNCA, but it may cause pathological aggregation of α-syn. The rs12411216 SNP is a causal SNP that affects *GBA* expression, and *GBA* activity was altered in the edited SH-SY5Y cells. *GBA* deficiency caused by decreased GBA enzymatic activity primarily leads to pathological accumulation of glycosphingolipids in lysosomes. Therefore, we hypothesized that the rs12411216 mutation would lead to a decrease in glucocerebrosidase (GBA protein, GCase) and enzyme activity, after which abnormally expressed GBA would affect the transport and degradation of a-Syn, thereby accelerating the formation of Lewy bodies and promoting the pathogenesis of PD (Fig. 5).

However, the current results were only based on knockout and replacement experiments on cell lines using CRISPR/Cas9-mediated genome editing methods. Evidence for the molecular genetics of PD-MCI is not currently available. A better explanation for the relationship between the rs12411216 mutation and GBA expression might be available if the brain tissue of an animal model for GCase enzyme activity could be determined. In our future research, we intend to construct an iPSC model (PD patient source) or a Parkinson’s disease mouse model involving an A53T and *GBA* rs12411216 double mutation to explore the relationship between rs12411216 and PD-MCI.

In summary, we showed that the distal regulatory SNP rs12411216 could affect the expression and enzymatic activity of the GBA gene and enhance α-Syn aggregation, indicating its strong relevance to the pathological progression of Parkinson’s disease. Overall, this study illustrated the relationship between *GBA* mutation and α-Syn aggregation and demonstrated that the rs12411216 SNP was a causative variant that could serve as a de novo biomarker for PD-MCI prognosis.

## Supplementary information

**Additional file 1.**

## Data Availability

Supplementary data are available online at *Molecular Brain*. All authors have reviewed the contents of the manuscript, approved its contents and validated the accuracy of the data.
