# Supplementary material for: Characterization of a pathogenic variant in GBA for Parkinson’s disease with mild cognitive impairment patients
Source: Mol Brain. 2020 Jul 8;13:102. doi: 10.1186/s13041-020-00637-x (PMC7346430; doi:10.1186/s13041-020-00637-x)
Supplement: Supplementary file 1 — Additional file 1. [file 13041_2020_637_MOESM1_ESM.docx]

**Other experimental details**

## DHS sequence (chr12: 131621602–131623539)

>hg19_dna range=chr1:155164045-155164995 5'pad=100 3'pad=100 strand=+ repeatMasking=none

AGATCGCGCCATTGCACTCCAGCCTGGGCGACAGAGCAAGACTCCGTCTC

AAAACAAAAAAGGGGGGGGCAGAGAGTAGGTGGGGTGACAGAATGGGATTTACGGACACAGAACTATCCTTTGGGGAGCAGAGTGGTATTGGGGACAGGTAGGCACGTAGCGGGGGGCAAAAGCACACGTGAGGTGCGTAATGCGCGACAGCCAAGAGGTGTGTGCGGCAGCAGTGCACGAGGGGAGGAAGCTATCCCAGGCCGCGGCAGCACGTGGAACTTGGGTCCCGCAGTCGCAGGCTGCGCTCTAGGCCGACGGAAGCAGTTCCGCCTGTATTCAGAAAACAGTTCTCAGCCCCAAAGACACAGGTTGGAAAGAACACTTTAAAAACACTTCGGCGAGGCACGGGATTGGAGCGCATCAGATCAGCAGGCGAGAGACCACCGCTCTCGCCCGCGCGCCGCGCGGCGCCCGAGGCGCAGCTTTGTGTGCGGCGAGACCCTGGGGCAGGCGCTGGGCGGGGTGGGGACATCTGACGTCAGCGCGCCCGGGAGCGCGGGCCGGGGAGGCGGGGAGCCCCCCAACTCGCAGGCCGGAGAAAACACGAGTAGCTAGGTGGGCGGCCCCAAACCTCAACTCCCGGCATTGCCAAGCAACAGCCATTCAGTTCGGTTGCTGGGACACGCGTCACCATGGCGACGGCTCCGCGCCGCGCAGTCTGAGTACTTAAAGAGCAAGCGCGCGCAACGCCCGGGCGTCGGAAAGCGGCCTTCTGGGACTCCGCAAACTCCCGTTCTCCCTCCCCCCTCCCCCTTGCCATTCCCTTTGCCTTCCGGGCGCCAAGTCTCTCTGGCCTCCAAGGTGAACCCAACTCCCCAGCGCGCCCCGGGTTCCCGGGTCGGGCCGGCTTCAGGCGGTGGGGAGCGGGATCCCGGGCCCCGGGCGGGCGGGAGGGACGGG

## Primer information used to test for mutations in SNP rs12411216

Forward Primer) GAGCGCATCAGATCAGCAGG

Reverse Primer) GGCAAAGGGAATGGCAAGG

## *GBA*-SNP replacement experiment donor DNA

CAGGCGAGAGACCACAGCTCTCGCCCGCGCGC

## qPCR primer

*β-actin*：Forward Primer）CATGTACGTTGCTATCCAGGC

Reverse Primer）CTCCTTAATGTCACGCACGAT

*GBA*：Forward Primer）CATCCGCACCTACACCTATGC

Reverse Primer）TGAGCTTGGTATCTTCCTCTGG

*SNCA*：Forward Primer）AAGAGGGTGTTCTCTATGTAGGC

Reverse Primer）GCTCCTCCAACATTTGTCACTT

## Biotin-labelled probe in EMSA

The biotin labels of these two probes both are at the 5' end

1)the wild-type SNP biotin-labelled probe (rs12411216: C)

AGAGACCACCGCTCTCGCCC

2) the mutant SNP biotin-labelled probe (rs12411216: A)

AGAGACCACAGCTCTCGCC

## DHS fragments and SNPs screened using bioinformatics analyses and found to be regulated in Parkinson’s disease.

**
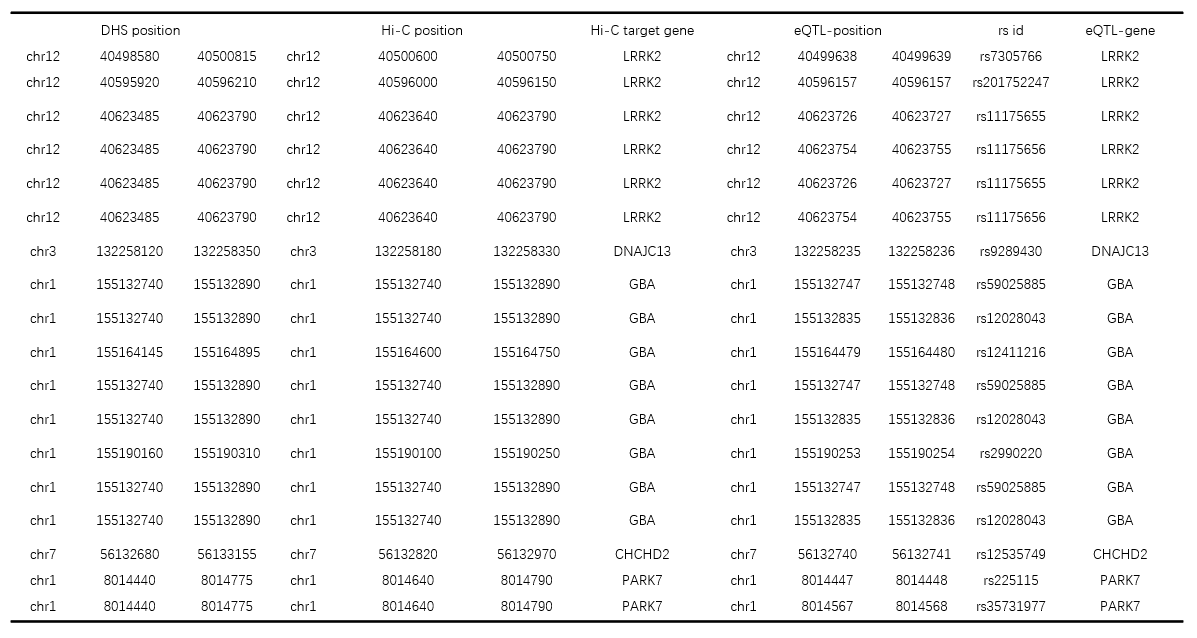
**
